# Novel Avian Influenza A(H5N8) Viruses in Migratory Birds, China, 2013–2014

**DOI:** 10.3201/eid2206.151754

**Published:** 2016-06

**Authors:** Li-Chen Zhou, Jing Liu, En-Le Pei, Wen-Jie Xue, Jia-Min Lyu, Yin-Ting Cai, Di Wu, Wei Wu, Yu-Yi Liu, Hui-Yu Jin, Yu-Wei Gao, Zheng-Huan Wang, Tian-Hou Wang

**Affiliations:** Laboratory of Wildlife Epidemic Diseases, Shanghai Key Laboratory for Urban Ecological Processes and Eco-Restoration, School of Life Sciences, East China Normal University, Shanghai, China (L.-C. Zhou, J. Liu, J.-M. Lyu, Z.-H. Wang, T.-H. Wang);; Shanghai Municipal Agency of Wildlife Conservation, Shanghai (E.-L. Pei, D. Wu, H.-Y. Jin); Chongming Dongtan National Nature Reserve, Shanghai (W.-J. Xue, W. Wu);; Jiuduansha Wetland National Nature Reserve, Shanghai, China (Y.-T. Cai) Wildlife Conservation Section, Shanghai Municipal Forestry Bureau, Shanghai (Y.-Y. Liu);; Research Center of Wildlife Disease, Key Laboratory of Jilin Province for Zoonosis Prevention and Control, Military Veterinary Research Institute of Academy of Military Medical Sciences, Changchun, China (Y.-W. Gao)

**Keywords:** subtype H5N8, migratory birds, influenza, highly pathogenic avian influenza virus, viruses, China

**To the Editor:** Novel highly pathogenic avian influenza (HPAI) A(H5N8) virus infections were first detected in poultry in eastern China in 2010 (*1*); the virus caused outbreaks in South Korea and Japan in 2014 (*2*) and reached Europe and North America by early 2015 (*3*–*6*). Phylogenetic analysis indicated that novel HPAI subtype H5N8 viruses might have originated in China and then circulated in East Asia countries and that the global geographic dissemination of this virus was strongly associated with the migration of wild birds (*7*). However, the role of migratory birds in the initial introduction and spread of novel H5N8 strains in China and other countries in the region is unclear. Shanghai, located at the Yangtze River estuary on the eastern coast of China, is a crucial stopover for migratory birds in East Asia. We report the presence of novel H5N8 strains from migratory birds sampled in Shanghai from October 2013 through December 2014.

A total of 26 novel H5N8 viruses were detected from migratory ducks and curlews captured and swabbed during their wintering period at the coastal wetlands of Shanghai. We collected 19 H5N8 viruses from 16 common teals (*Anas crecca*), 2 falcated ducks (*A. falcata*), and 1 spot-billed duck (*A. poecilorhyncha*) sampled in 2013 and 7 viruses from Eurasian curlews (*Numenius arquata*) sampled in 2014. Common teals were also found to be infected with subtype H5N1, detected by N1 gene fragments in 3 mixed-infection and 2 single-infection samples (Technical Appendix). Sequences from this study were deposited in GenBank (accession nos. KT936635–KT936716).

Homology BLAST (http://blast.st-va.ncbi.nlm.nih.gov/Blast.cgi) searches showed that H5 and N8 genes of 18 influenza A(H5N8) viruses in ducks had >98% similarity to H5N8 isolates W24 and 6D18 detected in poultry in Zhejiang Province (*2*), adjacent to Shanghai. The H5 gene in A/common teal/Shanghai/1108–1/2013(mixed) (PD1108-1) was 96% related to low pathogenicity avian influenza (LPAI) subtype H5N1 isolated from a European teal sampled in Russia in 2011 (GenBank accession no. KF462362). Of the 7 viruses from curlews, H5 and N8 isolates were closely related to isolates H68 and H297 from wild ducks reported in South Korea in early 2014 (*8*). Matrix genes of all novel subtype H5N8 viruses were closely related (95%–99%) to isolates from China (S11090, W24), Japan (156), and South Korea (Gochang1, S005) (online Technical Appendix Table).

Phylogenetic analysis of HPAI H5 descendants of A/goose/Guangdong/1/1996(H5N1) showed that clade 2.3.4.4 (*10*) H5N8 viruses fall into 2 distinct groups, closely related to group A (Buan2-like) and group B (Gochang1-like) (*8*). The hemagglutinin (HA) genes of the 18 subtype H5N8 viruses from ducks in 2013 shared a protease cleavage site motif of REKRRKR/GLF and the sequence cluster with H5N8 viruses from eastern China poultry (W24, 6D18) (*2*) and Korean group B isolates (Gochang1, H52) (*8*,*9*) to form group B. The HA genes from all 7 H5N8 isolates from curlews in 2014 had a protease cleavage site motif of RERRRKR/GLF and the sequence cluster, along with Korean group A (Buan2-like) (*8*,*9*), European (*3*,*4*), and North American (*7*) H5N8 lineage viruses to form group A. The HA from PD1108-1 had a cleavage site motif (RE–TR/GLF) characteristic of LPAI HA, and its sequence clustered with the Eurasian LPAI H5 lineage (Figure).

**Figure:**
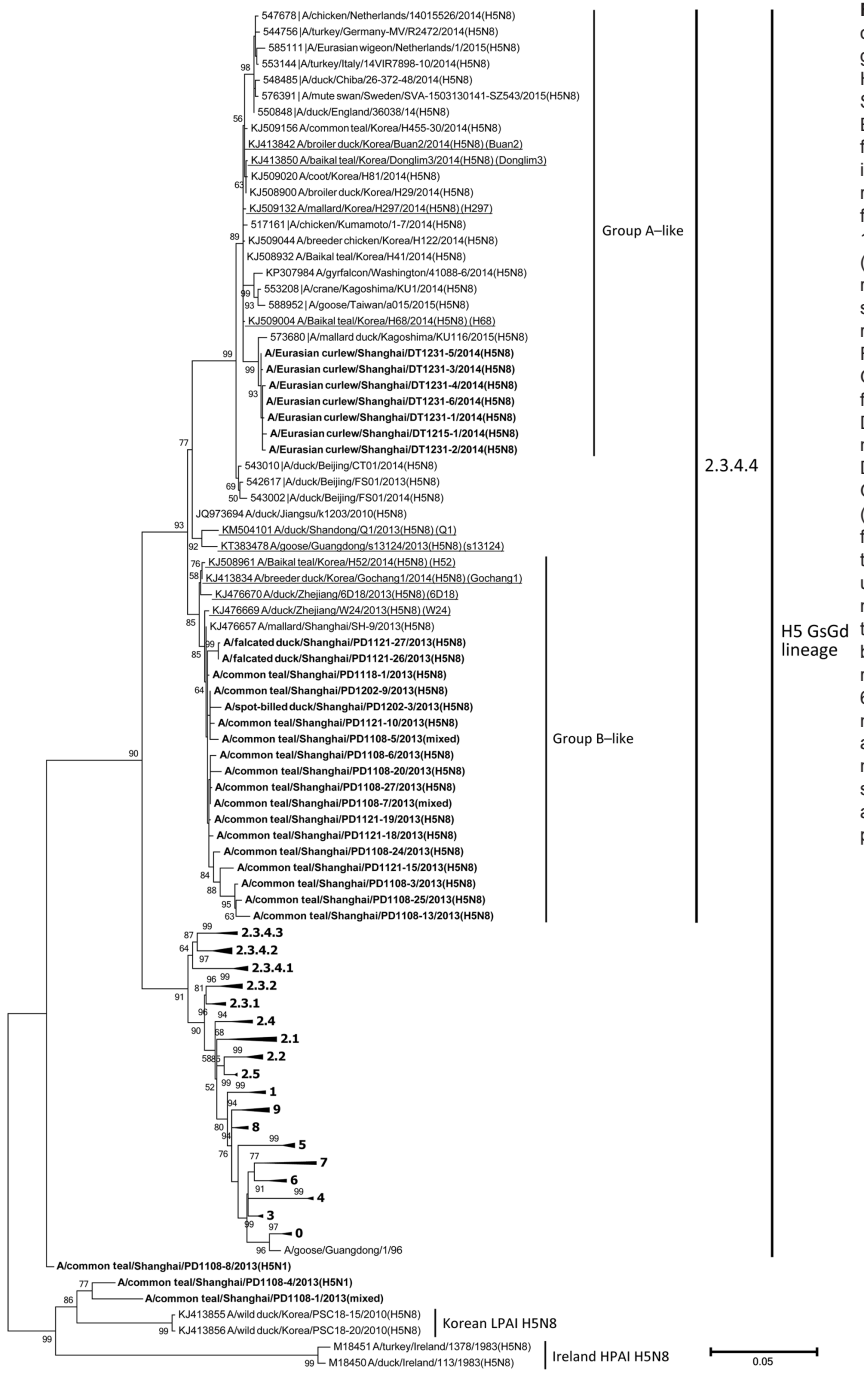
Phylogenetic tree of the hemagglutinin (HA) genes of influenza A subtype H5 viruses from wild birds of Shanghai, China, 2013–2014. Boldface indicates viruses from this study; representative isolates are underlined and referred to in abbreviated form in brackets. A total of 109 HA gene sequences (≥1,594 nt) were used for tree reconstruction. Representative strains and clades are recommended by WHO/OIE/FAO H5N1 Evolution Working Group and were retrieved from Influenza Virus Resource Database (http://www.ncbi.nlm.nih.gov/genomes/FLU/Database/select.cgi) and GISAID’s EpiFluTM Database (http://platform.gisaid.org/epi3/frontend). The phylogenetic tree was constructed by using the maximum likelihood method based on the general time reversible model with bootstrap analysis (100 replicates), by MEGA version 6 (http://www.megasoftware.net/). Bootstrap values ≥50% are shown. Scale bar indicates nucleotide substitutions per site. (See Technical Appendix.)

According to the sampling dates, the identification of the 18 group B H5N8 isolates from Shanghai was the earliest detection of HPAI H5N8 virus in wild birds in East Asia, before the first reported outbreak in South Korea in January 2014. Although poultry isolates from China obtained during the same period were phylogenetically clustered with group A (Figure), no group A viruses were detected in wild birds during the 2013–2014 wintering season in China. Notably, 2 of the group A Chinese poultry isolates (Q1 and s13124) have the HA cleavage site motifs of group B. Their topologically basal positions in group A (Figure) implied the connection between the 2 groups. Eurasian curlews are widely distributed in the Northern Hemisphere, including Europe, Siberia, Japan, the Korean Peninsula, and China (http://ibc.lynxeds.com/species/eurasian-curlew-numenius-arquata). Populations wintering in Shanghai have overlapped migratory routes and habitat distribution with duck species in East Asia (Shanghai Chongming Dongtan National Nature Reserve, unpub. data), which suggested possible transmission routes through overlapped habitats in their northern territory (breeding areas) or close contacts among these species. These data support the theory that asymptomatic migratory birds may have played a role in geographic dissemination of HPAI subtype H5N8 and facilitation of viral evolution and reassortment. Moreover, that HPAI subtypes H5N1 and H5N8 co-infected and co-circulated in migratory ducks suggests that rapid and active mutation and reassortment of H5 subtypes may take place in these hosts. Therefore, to monitor and then control the epidemics of H5 subtype viruses, it is urgent that more intensive surveillance be carried out in poultry and wild birds and that information be promptly shared among countries.

Technical Appendix A detailed description of the study materials and methods and a table showing genetic details of H5 viruses detected in migratory birds of Shanghai, China, 2013–2014.
