# Supplementary material for: Novel Avian Influenza A(H5N8) Viruses in Migratory Birds, China, 2013–2014
Source: Emerg Infect Dis. 2016 Jun;22(6):1121–3. doi: 10.3201/eid2206.151754 (PMC4880068; doi:10.3201/eid2206.151754)
Supplement: Technical Appendix — A detailed description of the study materials and methods and a table showing genetic details of H5 viruses detected in migratory birds of Shanghai, China, 2013–2014. [file 15-1754-Techapp-s1.pdf]

Article DOI: http://dx.doi.org/10.3201/eid2206.151754

Novel Avian Influenza A(H5N8) Viruses in Migratory Birds, China, 2013–2014

Technical Appendix

Materials and Methods

Wild Bird Surveillance and Sampling

Wild birds were captured and sampled with the permission and supervision of Shanghai Wild Life Conservation and Management Office. Swabs (1 oropharyngeal and 1 cloacal of each bird) were taken and transported in viral transport medium to the laboratory at 4°C for downstream molecular diagnosis within 6 hours.

Virus Detection

Viral RNA was extracted from viral transport medium by using MagMAX™ Pathogen RNA/DNA Kit (Applied Biosystems, Foster City, CA, USA) on a Magmax-96 Express (Applied Biosystems) according to the manufacturer's instructions. Real time reverse transcription-PCR was performed with matrix gene–specific probed primers to detect the presence of avian influenza viruses according to the protocol of the Chinese Center for Disease Control and Prevention (*1*). The results were further confirmed by sequencing the PCR products.

Subtype Identification and Gene Sequencing

RNA of viral-positive samples were reverse-transcribed by PrimeScript II Reverse Transcriptase Kit (TaKaRa, Biotechnology [Dalian] Co., Ltd, Dalian, China) according to the kit protocol. PCR amplifications were performed for subtyping of hemagglutinin (HA) and neuraminidase, and gene segments were amplified by using the previously published primers (*2*–

4) and primers designed in this study (on request). PCR products were gel-purified by using the QIAquick gel extraction kit (QIAGEN, Valencia, CA, USA) and were sequenced with the BigDye terminator kit (Applied Biosystems) on an ABI 3730 (Applied Biosystems). Sequences were assembled and edited with the Lasergene sequence analysis software package (http://www.dnastar.com/t-dnastar-lasergene.aspx). Primers for sequencing were similar to those used for amplification.

Phylogenetic Analysis

A phylogenetic tree was constructed of the HA genes of H5 viruses in wild birds of Shanghai, China, 2013–2014 (see text Figure). The viruses of this study are indicated in boldface. Representative isolates are underlined and referred to in abbreviation form in brackets. A total of 109 HA gene sequences (≥1,594 nt) were used for tree reconstruction. Representative strains and clades are recommended by WHO/OIE/FAO H5N1 Evolution Working Group and were retrieved from Influenza Virus Resource Database (http://www.ncbi.nlm.nih.gov/genomes/FLU/Database/select.cgi) and GISAID's EpiFluTM Database (http://platform.gisaid.org/epi3/frontend). The phylogenetic tree was constructed by using the maximum likelihood method based on the general time reversible model (best fit model: general time reversible + I + R, computed by jModelTest 2.1.4 (*5*,*6*) with bootstrap analysis (100 replicates), by MEGA v. 6 (*7*).Scale bar indicates nucleotide substitutions per site. Bootstrap values ≥ 50% are shown.

References

1. Chinese Center for Disease Control and Prevention. Chinese National Influenza Center Standard Operation Protocol. Beijing: The Center; 2007. p. 101–25.

2. Hoffmann E, Stech J, Guan Y, Webster RG, Perez DR. Universal primer set for the full-length amplification of all influenza A viruses. Arch Virol. 2001;146:2275–89. PubMed http://dx.doi.org/10.1007/s007050170002

3. Tsukamoto K, Ashizawa H, Nakanishi K, Kaji N, Suzuki K, Okamatsu M, et al. Subtyping of avian influenza viruses H1 to H15 on the basis of hemagglutinin genes by PCR assay and molecular determination of pathogenic. J Clin Microbiol. 2008;46:3048–55. PubMed http://dx.doi.org/10.1128/JCM.02386-07

4. Fereidouni SR, Starick E, Grund C, Globig A, Mettenleiter TC, Beer M, et al. Rapid molecular subtyping by reverse transcription polymerase chain reaction of the neuraminidase gene of avian influenza A viruses. Vet Microbiol. 2009;135:253–60. PubMed http://dx.doi.org/10.1016/j.vetmic.2008.09.077

5. Darriba D, Taboada GL, Doallo R, Posada D. jModelTest 2: more models, new heuristics and parallel computing. Nat Methods. 2012;9:772. PubMed http://dx.doi.org/10.1038/nmeth.2109

6. Guindon S, Gascuel O. A simple, fast and accurate method to estimate large phylogenies by maximum-likelihood. Syst Biol. 2003;52:696–704. PubMed http://dx.doi.org/10.1080/10635150390235520

7. Tamura K, Stecher G, Peterson D, Filipski A, Kumar S. MEGA6: Molecular Evolutionary Genetics Analysis version 6.0. Mol Biol Evol. 2013;30:2725–9. PubMed http://dx.doi.org/10.1093/molbev/mst197

Table. Genetic details of H5 viruses detected in migratory birds of Shanghai, 2013–2014*

		Closest related virus strain/nt identity				
Virus name	Collection date	HA (%)	NA (%)	MP (%)	HA cleavage site	Pathogenicity
A/Common Teal/Shanghai/PD1108-1/2013(mixed)	2013 Nov 8	Novo (96)	6D18 (99)	W24 (96)	RE- - - TR/GLF	Low
A/Common Teal/Shanghai/PD1108-3/2013(H5N8)	2013 Nov 8	w24 (98)	6D18 (99)	S11090 (95)	REKRRKR/GLF	High
A/Common Teal/Shanghai/PD1108-5/2013(mixed)	2013 Nov 8	w24 (99)	6D18 (99)	W24 (99)	REKRRKR/GLF	High
A/Common Teal/Shanghai/PD1108-6/2013(H5N8)	2013 Nov 8	w24 (99)	6D18 (99)	Gochang1 (99)	REKRRKR/GLF	High
A/Common Teal/Shanghai/PD1108-7/2013(mixed)	2013 Nov 8	w24 (99)	6D18 (99)	W24 (99)	REKRRKR/GLF	High
A/Common Teal/Shanghai/PD1108-13/2013(H5N8)	2013 Nov 8	w24 (98)	6D18 (99)	Gochang1 (99)	REKRRKR/GLF	High
A/Common Teal/Shanghai/PD1108-20/2013(H5N8)	2013 Nov 8	w24 (99)	6D18 (99)	Gochang1 (98)	REKRRKR/GLF	High
A/Common Teal/Shanghai/PD1108-24/2013(H5N8)	2013 Nov 8	w24 (99)	6D18 (99)	Gochang1 (99)	REKRRKR/GLF	High
A/Common Teal/Shanghai/PD1108-25/2013(H5N8)	2013 Nov 8	w24 (98)	6D18 (99)	Gochang1 (98)	REKRRKR/GLF	High
A/Common Teal/Shanghai/PD1108-27/2013(H5N8)	2013 Nov 8	w24 (99)	6D18 (99)	Gochang1 (98)	REKRRKR/GLF	High
A/Common Teal/Shanghai/PD1118-1/2013(H5N8)	2013 Nov 18	w24 (99)	6D18 (99)	W24 (98)	REKRRKR/GLF	High
A/Common Teal/Shanghai/PD1121-10/2013(H5N8)	2013 Nov 21	w24 (99)	6D18 (99)	Gochang1 (98)	REKRRKR/GLF	High
A/Common Teal/Shanghai/PD1121-15/2013(H5N8)	2013 Nov 21	w24 (99)	6D18 (99)	W24 (99)	REKRRKR/GLF	High
A/Common Teal/Shanghai/PD1121-18/2013(H5N8)	2013 Nov 21	w24 (99)	6D18 (99)	W24 (99)	REKRRKR/GLF	High
A/Common Teal/Shanghai/PD1121-19/2013(H5N8)	2013 Nov 21	w24 (99)	6D18 (99)	W24 (99)	REKRRKR/GLF	High
A/Falcated Duck/Shanghai/PD1121-26/2013(H5N8)	2013 Nov 21	w24 (99)	6D18 (99)	W24 (98)	REKRRKR/GLF	High
A/Falcated Duck/Shanghai/PD1121-27/2013(H5N8)	2013 Nov 21	w24 (99)	6D18 (99)	156 (95)	REKRRKR/GLF	High
A/Spot-billed Duck/Shanghai/PD1202-3/2013(H5N8)	2013 Dec 2	w24 (99)	6D18 (99)	W24 (99)	REKRRKR/GLF	High
A/Common Teal/Shanghai/PD1202-9/2013(H5N8)	2013 Dec 2	w24 (99)	6D18 (99)	W24 (99)	REKRRKR/GLF	High
A/Eurasian Curlew/Shanghai/DT1215-1/2014(H5N8)	2014 Dec 15	H68 (99)	H297 (99)	S005 (99)	RERRRKR/GLF	High
A/Eurasian Curlew/Shanghai/DT1231-1/2014(H5N8)	2014 Dec 31	H68 (99)	H297 (99)	S005 (99)	RERRRKR/GLF	High
A/Eurasian Curlew/Shanghai/DT1231-2/2014(H5N8)	2014 Dec 31	H68 (99)	H297 (99)	S005 (99)	RERRRKR/GLF	High

Virus name	Collection date	Closest related virus strain/nt identity HA (%)	NA (%)	MP (%)	HA cleavage site	Pathogenicity
A/Eurasian Curlew/Shanghai/DT1231-3/2014(H5N8)	2014 Dec 31	H68 (99)	H297 (99)	S005 (99)	RERRRKR/GLF	High
A/Eurasian Curlew/Shanghai/DT1231-4/2014(H5N8)	2014 Dec 31	H68 (99)	H297 (99)	S005 (99)	RERRRKR/GLF	High
A/Eurasian Curlew/Shanghai/DT1231-5/2014(H5N8)	2014 Dec 31	H68 (99)	H297 (99)	S005 (99)	RERRRKR/GLF	High
A/Eurasian Curlew/Shanghai/DT1231-6/2014(H5N8)	2014 Dec 31	H68 (99)	H297 (99)	S005 (99)	RERRRKR/GLF	High
A/Common Teal/Shanghai/PD1108-4/2013(H5N1)	2013 Nov 8	Novo (96)	/	W24 (97)	RE- - - TR/GLF	Low
A/Common Teal/Shanghai/PD1108-8/2013(H5N1)	2013 Nov 8	w24 (95)	/	MHC40-28 (97)	REKRRKR/GLF	High

*HA, hemagglutinin; MP, matrix protein; NA, neuraminidase; species: common teal (*Anas crecca*);fFalcated duck (*Anas falcata*); spot-billed duck (*Anas poecilorhyncha*); Eurasian curlew (*Numenius arquata*); sampling site: PD, Pudong eastern coastal wetlands; DT, Dongtan Bird Nature Reserve; homologue abbreviations: HA: novo, A/European teal/Novosibirsk/203/2011(H5N1)KF462362; w24, A/duck/Zhejiang/W24/2013(H5N8)KJ476669; H68, A/Baikal teal/Korea/H68/2014(H5N8)KJ509004; NA: 6D18,A/duck/Zhejiang/6D18/2013(H5N8)KJ476674; H297,A/mallard/Korea/H297/2014(H5N8)KJ509134; M: w24,A/duck/Zhejiang/W24/2013(H5N8)KJ476675; S11090, A/duck/Hunan/S11090/2012(H4N6)CY146551; Gochang1, A/breeder duck/Korea/Gochang1/2014(H5N8)KJ413837; 156, A/duck/Hokkaido/156/2006(H11N9)AB428725; S005, A/waterfowl/Korea/S005/2014(H5N8)KJ511815; MHC40-28, A/wild duck/Korea/MHC40-28/2010(H7N7)KC609942; / indicates no data.
